# Clinical quantitative cardiac imaging for the assessment of myocardial ischaemia

**DOI:** 10.1038/s41569-020-0341-8

**Published:** 2020-02-24

**Authors:** Marc Dewey, Maria Siebes, Marc Kachelrieß, Klaus F. Kofoed, Pál Maurovich-Horvat, Konstantin Nikolaou, Wenjia Bai, Andreas Kofler, Robert Manka, Sebastian Kozerke, Amedeo Chiribiri, Tobias Schaeffter, Florian Michallek, Frank Bengel, Stephan Nekolla, Paul Knaapen, Mark Lubberink, Roxy Senior, Meng-Xing Tang, Jan J. Piek, Tim van de Hoef, Johannes Martens, Laura Schreiber, on behalf of the Quantitative Cardiac Imaging Study Group

**Affiliations:** 1 Department of Radiology, Charité – Universitätsmedizin Berlin, Berlin, Germany; 2 Berlin Institute of Health and DZHK (German Centre for Cardiovascular Research) Partner Site, Berlin, Germany; 3 Department of Biomedical Engineering and Physics – Translational Physiology, Amsterdam University Medical Center, Amsterdam, Netherlands; 4 Division of X-Ray Imaging and CT, German Cancer Research Centre (DKFZ), Heidelberg, Germany; 5 The Heart Centre Rigshospitalet, Department of Cardiology and Radiology, University of Copenhagen, Copenhagen, Denmark; 6 MTA-SE Cardiovascular Imaging Research Group, Heart and Vascular Center, Semmelweis University, Budapest, Hungary; 7 Universitätsklinikum Tübingen, Radiologische Klinik, Diagnostische und Interventionelle Radiologie, Tübingen, Germany; 8 Biomedical Image Analysis Group, Department of Computing, Imperial College London, London, UK; 9 Institute of Diagnostic and Interventional Radiology and Department of Cardiology, University Hospital Zurich, University of Zurich, Zurich, Switzerland; 10 Institute for Biomedical Engineering, University and ETH Zurich, Zurich, Switzerland; 11 Department of Cardiovascular Imaging, School of Biomedical Engineering and Imaging Sciences, King’s College London, London, UK; 12 Physikalisch-Technische Bundesanstalt, Medical Physics and Metrological Information Technologies, Berlin, Germany; 13 Klinik für Nuklearmedizin, Medizinische Hochschule Hannover, Hannover, Germany; 14 Nuklearmedizinische Klinik und Poliklinik, Klinikum rechts der Isar der TU München, DZHK (German Centre for Cardiovascular Research), Partner Site Munich Heart Alliance, Munich, Germany; 15 Department of Cardiology, VU University Medical Center, Amsterdam, Netherlands; 16 Department of Surgical Sciences – Nuclear Medicine & PET, Uppsala University, Uppsala, Sweden; 17 Medical Physics, Uppsala University Hospital, Uppsala, Sweden; 18 Department of Cardiology, Royal Brompton Hospital London, London, UK; 19 Department of Bioengineering, Imperial College London, London, UK; 20 Heart Center, Amsterdam University Medical Center, Amsterdam, Netherlands; 21 Department of Cellular and Molecular Imaging, Comprehensive Heart Failure Center, Würzburg University Clinics, Würzburg, Germany

**Keywords:** Ischaemia, Magnetic resonance imaging, Computed tomography, Positron-emission tomography, Echocardiography, Radionuclide imaging

## Abstract

Cardiac imaging has a pivotal role in the prevention, diagnosis and treatment of ischaemic heart disease. SPECT is most commonly used for clinical myocardial perfusion imaging, whereas PET is the clinical reference standard for the quantification of myocardial perfusion. MRI does not involve exposure to ionizing radiation, similar to echocardiography, which can be performed at the bedside. CT perfusion imaging is not frequently used but CT offers coronary angiography data, and invasive catheter-based methods can measure coronary flow and pressure. Technical improvements to the quantification of pathophysiological parameters of myocardial ischaemia can be achieved. Clinical consensus recommendations on the appropriateness of each technique were derived following a European quantitative cardiac imaging meeting and using a real-time Delphi process. SPECT using new detectors allows the quantification of myocardial blood flow and is now also suited to patients with a high BMI. PET is well suited to patients with multivessel disease to confirm or exclude balanced ischaemia. MRI allows the evaluation of patients with complex disease who would benefit from imaging of function and fibrosis in addition to perfusion. Echocardiography remains the preferred technique for assessing ischaemia in bedside situations, whereas CT has the greatest value for combined quantification of stenosis and characterization of atherosclerosis in relation to myocardial ischaemia. In patients with a high probability of needing invasive treatment, invasive coronary flow and pressure measurement is well suited to guide treatment decisions. In this Consensus Statement, we summarize the strengths and weaknesses as well as the future technological potential of each imaging modality.

## Abstract (web summary)

Cardiac imaging has a pivotal role in the prevention, diagnosis and treatment of ischaemic heart disease. In this Consensus Statement, the authors summarize the use of SPECT, PET, MRI, echocardiography, CT and invasive coronary flow and pressure measurement, and describe the relative strengths and weaknesses of each modality.

## Introduction

International guidelines advocate noninvasive testing for patients with suspected ischaemia before proceeding with revascularization decision-making^1–4^. Noninvasive clinical cardiac imaging continues to undergo rapid evolution, focusing on quantitative perfusion technologies for the assessment of myocardial ischaemia and coronary flow. At present, imaging of myocardial ischaemia stands at a crossroads. During a European meeting on quantitative cardiac imaging, a bench-to-bedside-to-bench perspective was used to summarize the current status and future potential of myocardial ischaemia imaging from the viewpoint of basic scientists and clinical researchers. This approach created discussions, which led to this Consensus Statement on the main advantages and disadvantages of each imaging modality, a clinical consensus on the appropriateness for specific indications and a summary of the latest developments, which together provide a framework for future quantitative imaging of myocardial ischaemia.

## Pathophysiology considerations

**Fig. 1:**
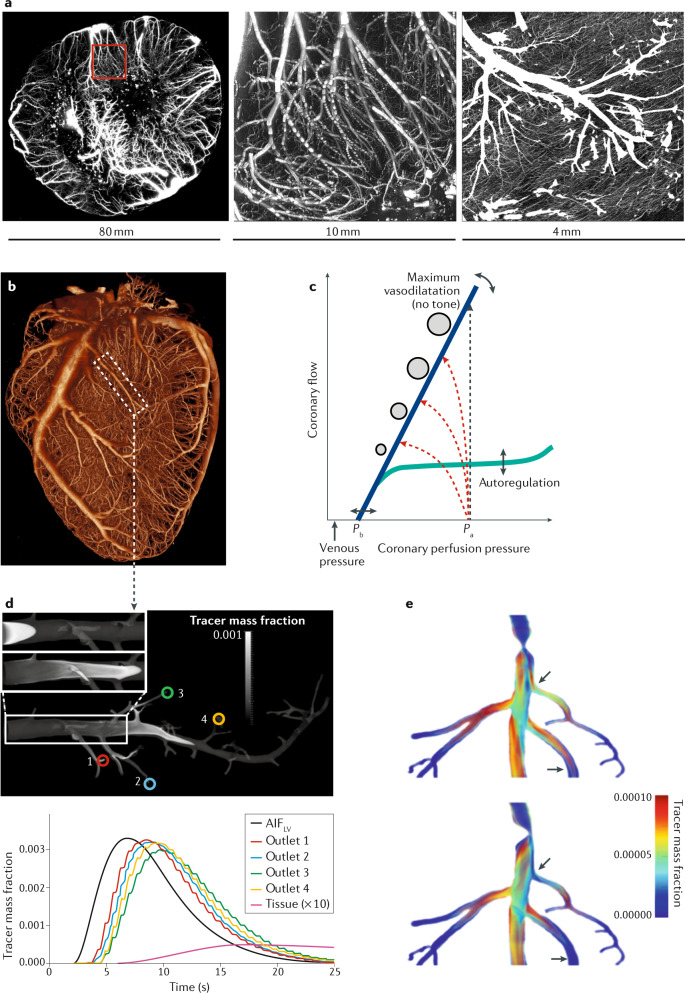
Interaction between coronary anatomy and physiology in relation to tracer distribution. **a** | Coronary arteries (left panel) penetrating the myocardium at the mid-ventricular level in a 3-mm-thick transverse section, as reconstructed from a 3D stack of cryomicrotome images^5^. The middle panel shows a magnified portion of the transmural microvascular network, as indicated by the red square in the left panel. Terminal arterioles perfusing the capillary bed are shown in the right panel. **b** | 3D reconstruction of coronary arteries and arterioles perfusing the heart muscle. **c** | Schematic illustration of the coronary pressure–flow relationship. Autoregulation maintains coronary flow at rest (green line) over a wide pressure range at a level adapted to oxygen consumption, whereas maximal flow without control (blue line) depends on coronary perfusion pressure. The zero-flow intercept incorporates collateral flow and depends on heart rate and ventricular wall tension^1^. In unobstructed vessels (black dashed line), flow increases with only negligible pressure loss at maximal vasodilatation. An epicardial stenosis induces progressive pressure loss with increasing flow (red dashed lines; stenosis severity increasing from top to bottom) and thereby raises minimal microvascular resistance. Stenosis resistance can be compensated at rest by lowering arterial tone, but limits maximal flow and compromises coronary flow reserve. **d** | Bolus-based perfusion methods such as MRI and CT typically obtain the arterial input function (AIF) from an easily visible anatomical region, such as the left ventricle (AIF_LV_). When tracer is transported along the epicardial vessels, the duration of the bolus increases (bolus dispersion). The upper part of the panel depicts a simulation of computational fluid dynamics that affect a vessel similar to that within the dashed rectangular region in part **b**^50^. Here, the assumption of a DOTA chelate-based tracer is made, which is injected quickly. For visualization purposes, simulations for the upper panel were made with a bolus 100 times shorter than that typically used in patients. See Supplementary Videos 1 and 2 for the dynamics. The lower panel shows the results from simulations of a real bolus, as used in humans. Similar bolus dispersion effects are expected for other tracers, depending on their diffusivity. The lower panel demonstrates that different regions (outlets 1–4) are exposed to slightly different AIFs (colours denote the different outlets in the upper part of the panel). If the observed bolus dispersion effects are not accounted for, a systematic underestimation of myocardial blood flow (MBF) of up to 45% can occur at rest, even in normal epicardial vessels without a stenosis^135^. The tissue curve is a typical concentration–time curve of the amount of tracer contained within a region of interest. For better visualization, the curve is scaled in amplitude by a factor of ten. The duration and shape of the curves depend on MBF. Tracer kinetic modelling of curves incorporates the AIF and results in a quantitative MBF value for that region. **e** | Tracer bolus broadening in a stenosed vessel. In addition to the bolus broadening in the normal epicardial vasculature, a stenosis increases the resistance and further disturbs bolus transport. Both effects depend on the shape and location of the stenosis (arrows), which results in additional bolus broadening and underestimation of MBF^185^. *P*_a_, aortic pressure; *P*_b_, extrapolated back pressure. Part **e** adapted with permission from ref.^185^, Wiley-VCH.

The coronary circulation comprises the epicardial conductance vessels (diameter 1–6 mm) feeding an extensive network of small vessels (diameter <300–400 μm) that penetrates the cardiac muscle tissue and is the site of regulation of myocardial blood flow (MBF; Fig. 1a,b). High-resolution 3D fluorescence cryomicrotome imaging^5^ has also revealed the existence of abundant small collateral vessels both between (intercoronary) and within (intracoronary) perfusion territories of major coronary arteries^6,7^. Importantly, blood supply via well-developed collaterals can reduce the area of myocardium at risk of ischaemia, which can be detected by all myocardial perfusion imaging techniques^8–10^. From a physiological perspective, microvascular resistance is closely adjusted at rest via integrated control mechanisms to maintain blood supply commensurate with cardiac workload and to compensate for pressure loss induced by an epicardial stenosis (autoregulation)^11–13^ (Fig. 1c). When microvascular resistance is minimized by potent vasodilators, such as adenosine, coronary flow becomes primarily dependent on perfusion pressure. Given that vessels without tone behave as elastic conduits, their diameters become pressure-dependent, and the minimized coronary resistance (at maximal vasodilatation; Fig. 1c) increases with decreasing perfusion pressure^14–16^. Regional myocardial perfusion in normal tissue is highly heterogeneous, attributable to vascular (asymmetric branching) and metabolic (O_2_ consumption) heterogeneity^17,18^. Transmural perfusion gradients and the microcirculation itself are further influenced by cardiac–coronary interaction with or without an epicardial obstruction. Compressive forces exerted by cardiac contraction render the subendocardium particularly vulnerable to ischaemia, especially at elevated heart rate or at low perfusion pressure distal to a stenosis^19–21^. The effects of epicardial obstruction, therefore, extend beyond the stenosis into the microcirculation^22^. The fundamental parameters for the assessment of myocardial ischaemia are regional absolute MBF (in units of millilitres per minute per gram of tissue) and relative perfusion, as well as relative parameters, such as myocardial perfusion reserve (MPR), which is the ratio of MBF under exercise-induced or pharmacologically induced stress to MBF at rest^22^.

## Ischaemia imaging modalities

The potential of noninvasive assessment of blood flow in the coronary microcirculation as a gatekeeper for invasive coronary angiography is the subject of a growing research effort. Important new insights have emerged regarding the clinical implications of the quantitative and qualitative assessment of myocardial ischaemia using the different myocardial perfusion imaging modalities, such as SPECT, PET, MRI, echocardiography and cardiac CT. In addition to summarizing the current clinical application of qualitative and quantitative ischaemia imaging, we also discuss the emerging possible use of artificial intelligence with deep learning for the detection and characterization of disease.

The unifying principle of all noninvasive techniques is that a contrast agent is injected into a peripheral vein. The contrast agent is transported to the heart and acts like a test substance (tracer) for blood and its transport through tissues. If the myocardium is normally perfused, the contrast agent is transported to the myocardium and can be detected with the use of noninvasive imaging modalities. If ischaemia is present, less contrast agent reaches the affected region and/or its wash-in or wash-out is delayed. This process is typically registered by a dynamic series of images that are sensitive to the specific contrast agent used.

Subsequent tracer kinetic modelling^16^ gives quantitative values of MBF and/or MPR. For this type of analysis, we need to know the time course of the contrast agent or tracer in the blood over time, that is, the arterial input function (AIF). The AIF is typically assessed either from the left ventricular cavity or by arterial blood sampling. From these principles, the AIF clearly accounts for variations in left ventricular function. However, collaterals cannot be accounted for and might introduce delayed wash-in^23^ and, on the basis of the mechanisms shown in Fig. 1d,e, might introduce systematic errors in the quantitative MBF or MPR values. Therefore, unlike catheter-based assessment and CT-based estimation of epicardial fractional flow reserve (FFR), the noninvasive ischaemia imaging techniques directly measure MBF and MPR, or at least provide a semiquantitative estimate. Some of these techniques allow the additional assessment of parameters beyond MBF and MPR and are used in further analysis by modern methods, such as machine and/or deep learning^24–27^. The common goal is to guide patient management and to provide the requisite justification for invasive coronary angiography and intracoronary haemodynamic measurements. Importantly, no single imaging technology can currently provide all measures of coronary circulation, and all imaging techniques have specific advantages and disadvantages while also providing different assessments of the coronary vascular components using anatomical or functional imaging (Table 1).

**Table 1:** Technical comparison of tools for the assessment of myocardial ischaemia

Parameter	SPECT	PET	MRI	Echocardiography	CT	Invasive coronary flow and pressure measurement
***General***						
Ionizing radiation use	Yes (radiopharmaceutical)	Yes (radiopharmaceutical)	No	No	Yes (X-rays)	Yes (X-rays)
Stressor	Exercise or vasodilator agents	Exercise or vasodilator agents	Mainly vasodilator agents	Exercise or vasodilator agents	Mainly vasodilator agents	Vasodilator agents
Contrast agent or tracer	^99m^Tc-sestamibi or ^99m^Tc-tetrofosmin	^15^O, ^82^Rb, ^13^NH_3_ or ^18^F-flurpiridaz	Gadolinium-based	Microbubbles	Iodine-based	None
Contrast agent or tracer distribution	Intracellular	Freely diffusible: intravascular, extracellular and intracellular	Intravascular and extracellular	Intravascular	Intravascular and extracellular	NA
Type of measurement	Static; dynamic feasible with new cameras	Dynamic bolus or static	Dynamic bolus	Clearance–reperfusion	Dynamic bolus or static	Pressure and/or flow velocity
Linear relationship between blood flow and tracer	No (underestimation at high flow)	Yes	No	Yes	No	NA
Linear relationship between tracer and image signal	Yes (radiopharmaceutical)	Yes	No	Yes	Yes	NA
Contrast-to-noise ratio	High	Low	High	High	Low	NA
Temporal resolution (acquisition time per frame)	10 s	1–5 s	Approximately 100–200 ms	Approximately 4–50 ms (20–250 frames per s)	Approximately 150–200 ms	NA
Spatial resolution (image analysis voxel size)	10 × 10 × 10 mm³	4 × 4 × 4 mm³	1 × 2 × 6–8 mm³	1 × 1–3 × 3–6 mm³ (spatially varying)	0.5 × 0.5 × 6–8 mm³	NA
Isotropic left ventricle coverage	Yes	Yes	No	No	Yes	NA
***Technical challenges***						
Stenosis quantification	NA	NA	Spatial resolution	NA	Beam hardening	Variability and projections
Coronary haemodynamic assessment	NA	NA	Spatial resolution	NA	Flow assumptions	Costs and availability
Ischaemia quantification	Spatial and temporal resolution	Spatial and temporal resolution	Contrast agent signal nonlinearity	Intravascular contrast agent	Contrast agent dynamics, noise and dose	NA
***Types of coronary vascular component assessment by each imaging modality***						
Epicardial conductance vessel (>500 μm)	Functional assessment	Functional assessment	Functional assessment	Functional assessment	Anatomical and functional assessment	Anatomical and functional assessment
Resistance vessels and arterioles (<500 μm)	Functional assessment	Functional assessment	Functional assessment	Functional assessment	Functional assessment	NA
Capillaries	Functional assessment	Functional assessment	Functional assessment	Functional assessment	Functional assessment	NA
Endocardial–epicardial flow ratio	NA	NA	Yes	NA	Yes	NA
Collaterals	Yes (but with limitations)	Yes	Yes	Yes	Yes	Yes
Contraction–flow relationship	NA	NA	Yes	Yes	Yes	Yes
***Advantages and disadvantages***						
Advantages	Wide availability and quantification now also possible in patients with a high BMI	Technically best-suited test for ischaemia quantification; novel tracers and small cyclotrons have become available	Assessment of function, perfusion and viability without ionizing radiation	Availability at the bedside for analysis without ionizing radiation	Quantitative scale and high spatial resolution for coronary stenosis and plaque analysis	Immediate treatment opportunity during the same procedure
Disadvantages	Low image quality owing to attenuation	Limited availability (available in specialized centres only)	Limited coronary stenosis analysis and quantification challenges	No coronary artery stenosis analysis and quantification challenges	Radiation dose, low contrast-to-noise ratio and quantification challenges	Limited use in patients without acute presentation

Consensus ratings were performed using a Delphi process with ratings by 20 investigators (six cardiologists, four radiologists, one dual cardiologist–radiologist, one nuclear medicine physician and eight methodologists). NA, not applicable.

## Technical characteristics and challenges

Major practical challenges for the assessment of myocardial ischaemia using the methods under consideration include the following:

- SPECT: radiation dose, spatial resolution and limited quantification of myocardial perfusion^28^.
- PET: lower spatial resolution of quantitative ^15^O-water PET than with myocardial CT or MRI perfusion imaging and limited visual assessment^29,30^.
- MRI: increased prevalence of pacemakers^31^ and limited spatial coverage of the left ventricle^32^.
- Echocardiography: common presence of noise and artefacts, lack of reproducibility, variable image quality and time-consuming manual analysis^33^.
- CT: limited temporal resolution, presence of beam and scatter artefacts, radiation dose, and low contrast-to-noise ratios^34–37^.
- Invasive FFR: diagnostic and prognostic characteristics, complexity of the procedure and its limited uptake in clinical practice^38,39^.

From technical and pathophysiological perspectives, numerous aspects need to be considered for the quantification of myocardial ischaemia. The tracer distribution characteristics vary widely between imaging techniques. MRI and CT tracers enter the extravascular extracellular space (interstitial space); nuclear tracers can also enter the intracellular space or even bind within the myocyte (most SPECT and PET tracers, except ^15^O-water) (Table 1). By contrast, microbubbles used for echocardiography remain in the intravascular space. On the basis of the different tracer characteristics, the agreement between the numerical results obtained with the different techniques varies^40–42^. Even microspheres, which are the experimental gold standard of perfusion, can distribute in a heterogeneous manner when the analysed region becomes small, possibly reflecting technical limitations and/or physiological variations in regional flow^43^. A nonlinear relationship exists between MBF and tracer uptake in SPECT and most PET tracers except ^15^O-water. Both MRI and CT use contrast agents that extravasate from the intravascular to the extravascular space in a fashion that is nonlinearly dependent on blood flow^44^. In addition, obtaining a linear relationship between MRI tracer concentration and signal intensity is difficult because of the complexity of MRI contrast mechanisms^45,46^. Accurately measuring the AIF is challenging with SPECT^47^, PET^48^, MRI^49,50^ and CT^51^ (Fig. 1c,d), which can result in biased measurements of MBF (Fig. 1e). Tracer kinetic modelling of MBF needs to account for these differences between modalities.

## Methods of consensus

The emerging imaging concepts and their challenges matter not only to cardiologists, radiologists and nuclear medicine physicians but also to non-specialists. As the field has become very complex, it deserves a comprehensive and up-to-date expert consensus. Experts in the cardiac imaging modalities from seven European countries, with a balance of clinical disciplines (cardiology, nuclear medicine and radiology) and basic scientists, were involved in the Quantitative Cardiac Imaging meeting. This Consensus Statement is not, therefore, endorsed by a single specialist society, but is driven by scientists from all disciplines collaborating to provide a multidisciplinary consensus. The content and recommendations in this article emerged from discussions among our diverse team, which aimed to overcome political and professional interests to produce balanced consensus recommendations. In a subsequent real-time Delphi process, the technical parameters of each modality were summarized by consensus (Table 1).

All the authors participated in the Delphi process and judged all imaging modalities, to result in a consensus across modalities. The consensus process was performed after all lectures were given and discussed during the Quantitative Cardiac Imaging meeting and was organized into technical and clinical consensus processes. During the technical consensus process (Table 1), a total of 20 authors participated (six cardiologists: K.F.K., A.C., P.K., R.S., J.J.P. and T.v.d.H.; four radiologists: M.D., K.N., R.M. and F.M.; one dual cardiologist–radiologist: P.M.-H; one nuclear medicine physician: F.B.; and eight methodologists: M.S., M.K., S.K., T.S., S.N., M.L., J.M. and L.S.). Technical assessments by every participant for all items were made transparent to all participants to allow real-time evaluation and reconsideration of his or her own judgements for the Delphi consensus process.

**Fig. 2:**
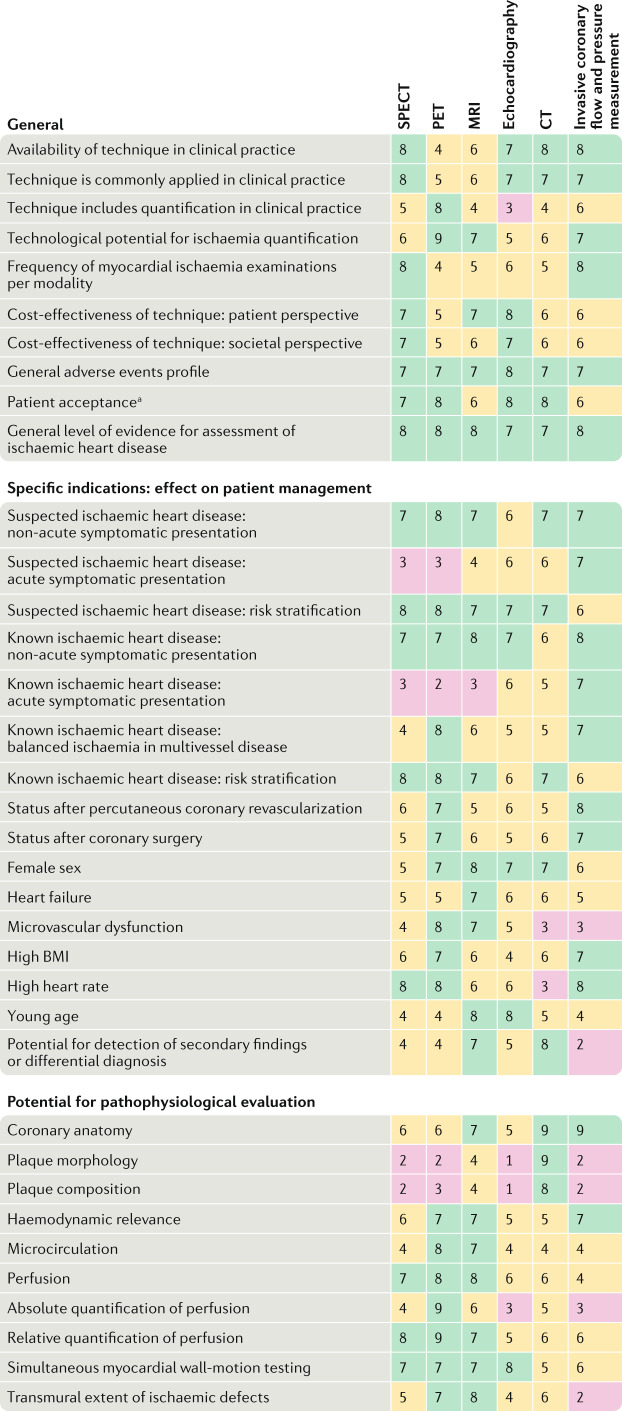
Clinical characteristics and appropriateness of myocardial ischaemia assessment tools for different patient scenarios. Consensus ratings were on a scale of 1–9, with 1–3 being inappropriate, 4–6 being uncertain and 7–9 being appropriate. A total of 16 investigators participated. A Delphi clinical consensus process was used, with ratings by eight participants (four cardiologists, two radiologists, one nuclear medicine physician and one methodologist). A separate development team of eight investigators (one dual cardiologist–radiologist, two radiologists and five methodologists) defined the questions and categories shown in the table but did not participate in the clinical appropriateness rating. ^a^Studies discussed during the meeting showed higher patient acceptance for CT than for SPECT, MRI or invasive testing^186–188^.

For the clinical consensus process (Fig. 2), a total of 16 authors participated and were divided into a development team and a clinical appropriateness team. The development team consisted of eight investigators (one dual cardiologist–radiologist: P.M.-H.; two radiologists: K.N. and F.M.; and five methodologists: M.S., T.S., A.K., J.M. and L.S.), who did not participate in the clinical appropriateness rating but defined the questions and categories used in the clinical consensus. The clinical appropriateness team consisted of eight investigators providing clinical consensus ratings for all methods and all clinical scenarios (four cardiologists: K.F.K., A.C., R.S. and J.J.P.; two radiologists: M.D. and R.M.; one nuclear medicine physician: F.B.; and one methodologist: M.L.). Judgements from each team member were fully taken into consideration, displayed in a transparent fashion during the real-time Delphi process and averaged at the end of the assessment to produce final appropriateness ratings (ranging from 1 to 9) for all imaging tests in all clinical settings.

Moreover, in this Consensus Statement, we avoided giving the final verdict on a ‘competition between the methods’, but instead presented the potential relative merits of the methods that are currently available. Differences of opinion will always exist, which cannot entirely be avoided, but our cross-disciplinary Delphi process represents a solid approach to the clinical question. This Delphi process helped to define appropriate clinical indications as well as some of the directions for future technical developments to overcome challenges of SPECT, PET, MRI, echocardiography, CT and invasive coronary flow and pressure measurements. This Consensus Statement also describes major trends and breakthroughs relevant to patients with cardiac disease for all six imaging modalities in the sections below. Although we attempt to give a balanced overview of all the available imaging modalities using the Delphi consensus process, region-specific or institution-specific availability or expertise might increase or decrease the relative utility of a particular modality.

## SPECT

### Role in assessment of ischaemic heart disease

SPECT has been the centrepiece of clinical cardiovascular imaging for decades. SPECT-based evaluation of the extent of myocardial ischaemia as a percentage of the left ventricular myocardium, together with concomitantly measured left ventricular function and volumes, is well established for the diagnosis of myocardial ischaemia^1^. SPECT not only has incremental prognostic value over clinical assessment alone but is also important in guiding therapy. The current clinical paradigm that the degree of ischaemia defines the individual risk, which in turn should be used for risk-based decision-making on revascularization or optimized medical therapy, is based on large registries of SPECT perfusion imaging^52^. Of note, no studies have compared outcomes when therapy is decided on the basis of SPECT versus an alternative test. As SPECT measures any perfusion to the myocyte with a tracer that is bound in the mitochondria, and owing to the temporal resolution, a distinction between primary-vessel and collateral flow cannot be made.

### Quantitative assessment of pathophysiology

**Fig. 3:**
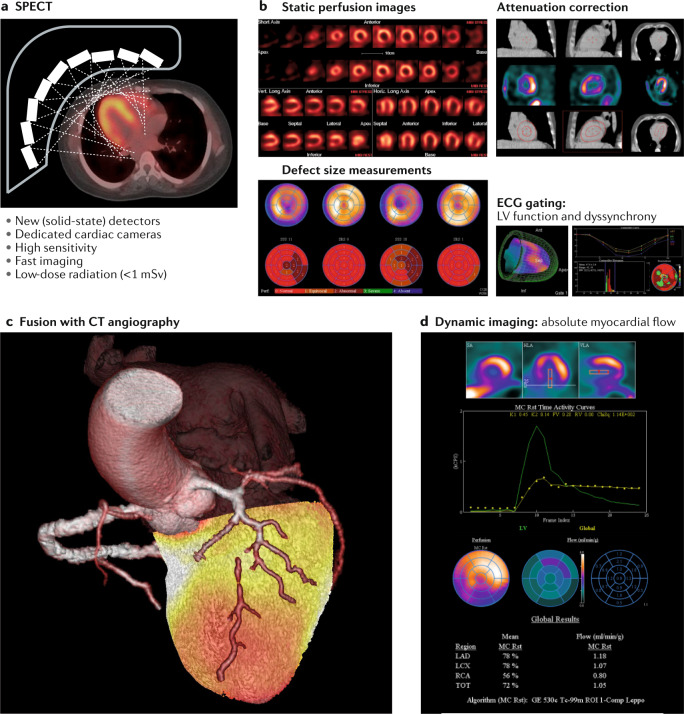
SPECT for quantification of myocardial ischaemia. **a** | Design of a new, dedicated cardiac SPECT camera with static solid-state detectors focused on the heart, with the major characteristics summarized below the image. **b** | Representative images of current state-of-the-art SPECT measurements, including static images along the cardiac axes (top left), polar maps and segmental scores for quantification of defect size (bottom left), quality-control screens for CT-based attenuation correction of SPECT images (top right) and volumetric information on left ventricular (LV) function and synchrony from electrocardiogram (ECG)-gated images (bottom right). **c** | Advanced methodology for 3D fusion of a SPECT dataset with coronary CT angiography, enabling localization of ischaemia to a coronary artery territory. This image shows anterior wall ischaemia resulting from chronic occlusion of the left anterior descending coronary artery. **d** | Advanced analysis of dynamic SPECT images for absolute quantification of myocardial blood flow, including region-of-interest placement for myocardium and blood pool (top), derivation of time–activity curves (middle) and parametric display of flow parameters derived from compartmental modelling (bottom), including correction for tracer-specific nonlinear flow and extraction (see Fig. 4a).

Detailed technical characteristics of SPECT are listed in Table 1. Technical evolution has occurred, with the aim of maximizing sensitivity to reduce both the amount of injected radiopharmaceuticals and the acquisition time. These advances have been paralleled by a shift towards increased BMI in the patient population. Solid-state detector technology based on cadmium–zinc–telluride (CZT) has been central to the technical advances in SPECT imaging. Small semiconductor detectors allow fully digital acquisition schemes and obviate the need for heavy sodium iodide crystals and large photomultipliers, thereby enabling compact camera designs (Fig. 3a). The high sensitivity (84%) of recent CZT SPECT systems and a specificity of 69% to detect obstructive coronary artery disease^53^ has enabled dynamic imaging to assess the kinetics of perfusion tracers in blood and the myocardium. This information allows compartmental modelling to delineate absolute MBF and coronary flow reserve (CFR) by SPECT. Recent work has shown the feasibility of MBF quantification with CZT SPECT and has provided validation against the microsphere gold standard in an experimental model^54^ and in humans^55^ compared with PET. Clinically implementing this approach with SPECT protocols has the potential to provide incremental diagnostic and prognostic value for ischaemia assessment over the previous standard of relative regional perfusion defect measurement^56,57^, because it can detect conditions that affect the entire myocardium, such as severe ischaemia in multivessel disease or microvascular dysfunction (mostly in the subendocardium), with the caveat of the limited spatial resolution of SPECT. Therefore, integrating measures of transient post-ischaemic dilatation further improves the diagnostic accuracy and can be calculated in a highly automated fashion^58^.

### Indications and clinical applications

SPECT is most commonly applied for myocardial perfusion imaging. Clinical indications for SPECT are listed in detail in Fig. 2. Major advantages of dedicated cardiac SPECT cameras include superior sensitivity and resolution while allowing radiation dose reduction across a range of patient conditions, as well as allowing the imaging of patients with challenging features, such as a high BMI^59,60^. Compared with regular SPECT, CZT systems increase the effective sensitivity by fourfold to tenfold, and also provide higher spatial and energy resolution^59,60^. Accordingly, high-quality images are obtained with shorter acquisition times and/or a smaller injected dose of the radiopharmaceutical, which is beneficial for various groups of patients with complicated disease. Several multicentre studies support the usefulness as well as the diagnostic and prognostic value of CZT-based myocardial perfusion SPECT^28^. Although early work with these novel systems focused on the reduction in imaging time from 15–30 min with standard SPECT down to 2–3 min with CZT SPECT using a standard amount of injected radioactivity^61,62^, the aim of subsequent work was to reduce the amount of injected radioactivity to minimize patient exposure to ionizing radiation. A recent multicentre trial suggests that CZT imaging can be completed with an effective dose of 1 mSv at an image quality that is still superior to that obtained with standard SPECT^63^.

### Future developments

Commercially available software tools for SPECT analysis can be readily implemented on every acquisition system, allowing reproducible measurement of perfusion defect sizes and ventricular function (Fig. 3b). These tools also enable registration and fusion of SPECT datasets with coronary CT angiography for improved prediction of long-term outcomes^64^ (Fig. 3c) and the latest developments provide options for the absolute quantification of MBF from dynamic datasets (Fig. 3d) and the integration of artificial intelligence techniques^24,65^. Future work will define how this comprehensive toolbox of quantitative parameters is best utilized in the work-up of myocardial ischaemia for guidance of targeted coronary interventions of the correct coronary artery, for the selection of coronary artery bypass grafting versus stenting versus optimal medical therapy alone, and for serial monitoring of the effects of therapy.

In summary, cardiac SPECT has advanced through the adoption of solid-state detector-equipped dedicated cameras as the new standard. This implementation enables fast assessment of myocardial perfusion and function with exposure to a low dose of radiation, and includes diagnostic and prognostic measures of relative regional ischaemia that are well established and quantitative. The next phase will include the quantification of MBF and CFR. The techniques are readily adopted from PET as the reference standard and can be translated to the field of SPECT owing to the increased sensitivity and the improved temporal resolution of the new cardiac camera systems. On the basis of its characteristics, SPECT is the primary clinical method for the assessment of myocardial ischaemia, and the latest technological advances now also allow robust imaging of patients with challenging features, such as those with a high BMI.

### Key points for SPECT

- SPECT is most commonly used in the clinic for myocardial perfusion imaging.
- Challenges of SPECT include radiation dose and limited quantification of myocardial perfusion^28^.
- Dedicated cardiac SPECT cameras have emerged, with improved sensitivity and resolution at lower radiation doses and in patients with challenging features (including those with a high BMI)^59,60^.
- Quantitative measures of the perfusion defect size have become more widely available as diagnostic and prognostic markers with the introduction of solid-state detector CZT technology.
- Dynamic imaging enables compartmental modelling and provides absolute measures of myocardial ischaemia, thereby bringing SPECT closer to PET as the reference standard.

## PET

### Role in assessment of ischaemic heart disease

**Fig. 4:**
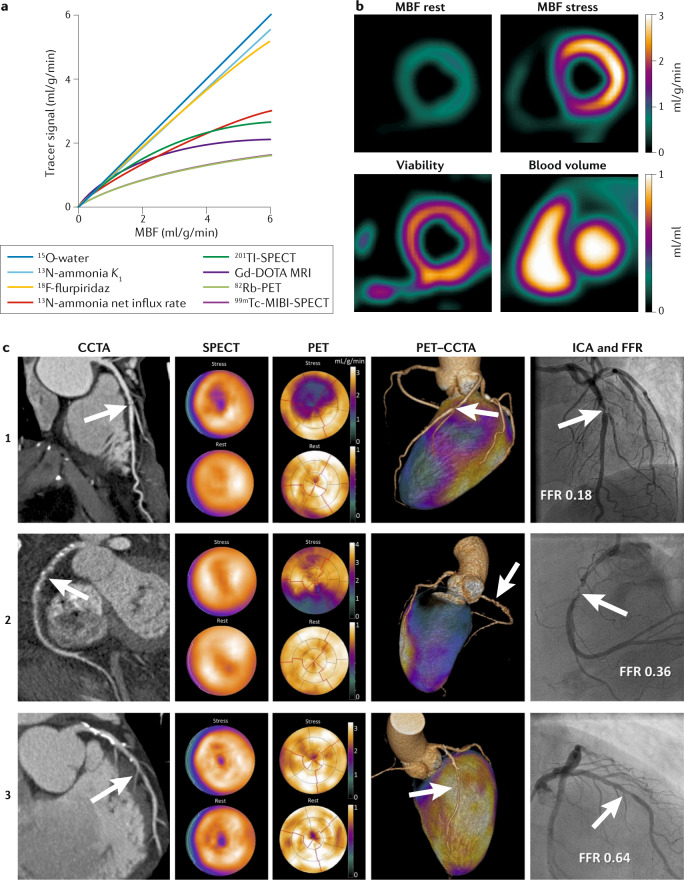
PET for quantification of myocardial ischaemia. **a** | Tracer signal versus myocardial blood flow (MBF), showing a constant 100% extraction for ^15^O-water, an almost 100% extraction for ^13^N-ammonia *K*_1_ and ^18^F-flurpiridaz, and a substantial roll-off phenomenon, resulting in underestimation of MBF that increases with MBF, for ^13^N-ammonia (when quantified using its net influx rate), ^201^Tl-SPECT, Gd-DOTA dynamic contrast-enhanced (DCE) MRI, ^82^Rb and ^99m^Tc-MIBI-SPECT^66,189–192^. Note that the ^82^Rb and ^99m^Tc curves overlap. **b** | MBF images at rest and during stress, myocardial viability and blood volume from a single rest–stress ^15^O-water protocol. This protocol can be used to assess ventricular volumes and ejection fraction. **c** | Example images from the PACIFIC trial showing coronary CT angiography (CCTA), SPECT, PET and invasive coronary angiography (ICA) images, with corresponding fractional flow reserve (FFR), in three patients (numbered 1–3) with coronary artery stenosis (arrows).

PET is an inherently quantitative imaging technique that allows the accurate measurement of radioactivity concentrations in vivo, making PET the primary test for patients with multivessel disease to confirm or exclude balanced ischaemia. Currently, four different PET tracers are used clinically for the assessment of MBF: ^82^Rb, ^13^N-ammonia, ^15^O-water and ^18^F-flurpiridaz^66^. On the basis of close to 100% extraction, ^15^O-water PET is considered the clinical gold standard for perfusion quantification given that the image signal is directly proportional to tracer concentration, and tracer kinetic modelling is well established. All other tracers show a varying decrease in extraction with increasing flow, requiring correction to derive MBF (Fig. 4a). Because of their short half-lives, ^82^Rb and ^15^O-water allow the completion of rest–stress protocols within 30 min, with a total radiation dose that is lower than that of SPECT, although new SPECT technologies considerably reduce the radiation dose, as described above.

### Quantitative assessment of pathophysiology

Detailed technical characteristics of PET are listed in Table 1. Because ^15^O-water is freely diffusible, no simple static uptake PET images are available for visual assessment, and images of perfusion defects are sometimes grainy and difficult to interpret. Therefore, quantification is needed for clinical assessment using ^15^O. With ^82^Rb, ^13^N-ammonia and ^18^F-flurpiridaz, perfusion defects are visualized in a similar way to SPECT, but absolute MBF measurements can be provided using tracer kinetic modelling and correction for limited extraction. However, absolute measures remain variable, and a lack of standardization exists between systems. The complexity of quantitative data analysis has long hampered the clinical feasibility of ^15^O-water PET. However, advances in scanner technology, image reconstruction methods and data analysis during the past decade have enabled nearly automated and fast computation of parametric images showing MBF at the voxel level and have facilitated the use of ^15^O-water in the clinical setting^29,30^. Another disadvantage of the lack of uptake images using ^15^O-water is that myocardial function and volumes cannot easily be quantified. Estimating ejection fractions from gated first-pass images has been shown to correlate well with values derived from MRI^30,67^. An advantage of the free diffusibility of ^15^O-water is that it allows the calculation of the perfusable tissue fraction, which is the ratio between uptake and clearance rate constants. Normalizing this parameter for the anatomical tissue fraction gives the perfusable tissue index, which is a marker of myocardial viability^68,69^. Therefore, MBF, viability and functional parameters can now be determined from a single ^15^O-water PET scan (Fig. 4b).

### Indications and clinical applications

Myocardial perfusion PET is increasingly being used for the evaluation of patients with known or suspected myocardial ischaemia. Clinical indications for PET are listed in detail in Fig. 2. The vast majority of studies have been conducted with static uptake images of ^82^Rb and ^13^N-ammonia. In a pooled analysis of these studies, weighted sensitivity, specificity, negative predictive value and positive predictive value were 91%, 86%, 81% and 93%, respectively^70^. However, almost all these studies compared PET with invasive coronary angiography without invasive coronary flow or pressure measurement^71^. For such indirect detection of obstructive epicardial stenosis, PET is as accurate as CT or MRI perfusion imaging^72,73^. In addition, mounting evidence indicates that quantitative analysis of PET is superior to static-uptake image grading^74–76^. Even more compelling are the observations that hyperaemic MBF quantification outperforms PET-derived CFR in the diagnosis of obstructive coronary artery disease, highlighting the potential of stress-only protocols^77,78^. Unfortunately, reported thresholds of what should be considered pathological hyperaemic MBF or CFR are not uniform across PET tracers^22^. Cut-off values seem to be, at least in part, related to tracer kinetics and should not be considered interchangeable.

In a contemporary study, PET had higher accuracy (85%) than coronary CT angiography (74%) or SPECT (77%) for the diagnosis of myocardial ischaemia when taking FFR as a reference standard^40^ (Fig. 4c). The comparative accuracy of PET versus CT or MRI perfusion, which have higher spatial resolution, remains to be determined. In terms of prognosis, analogy to large-scale SPECT databases exists^79^. The extent and severity of (reversible) perfusion defects documented with PET hold strong prognostic information beyond that obtained from traditional cardiovascular risk factors^22^. PET-derived CFR has shown added prognostic value for the identification of at-risk women with more frequent non-obstructive coronary artery disease and potential microvascular dysfunction^57^. Of particular interest is that apparently normal perfusion images with a homogeneous tracer distribution can be reclassified on the basis of diffusely blunted hyperaemic MBF or CFR; several studies have shown that this subset of patients is at increased risk of future cardiac events^80–82^.

### Future developments

Both ^15^O-water and ^13^N-ammonia require the presence of an on-site cyclotron as a high upfront investment because of the short half-lives of both tracers (2 min and 9 min, respectively). By contrast, ^82^Rb (with a half-life of 1.3 min) is produced by a generator that has to be replaced on a monthly basis, which confers a high monthly cost but a low upfront investment. So-called baby cyclotrons have lower cost and are aimed specifically at the production of ^13^N or ^15^O and, therefore, also require lower energies and considerably less radiation shielding than common PET cyclotrons. This feature greatly increases the possibility of using ^15^O-water and ^13^N-ammonia in hospitals without PET chemistry facilities. ^18^F-flurpiridaz is a novel tracer that has shown higher accuracy than SPECT^83^ and also promises quantification using direct parametric reconstruction^84^. Furthermore, machine learning used for the integration of variables has great potential in clinical practice for identifying patients with myocardial ischaemia and increased risk of events^25^.

In summary, cardiac PET is the reference standard for the quantification of perfusion imaging. PET using parametric images showing MBF at the voxel level is now available in routine clinical practice, and PET also allows the derivation of the perfusable tissue index, which is a marker of myocardial viability, thereby expanding the clinical utility of this modality. On the basis of its characteristics, PET is well suited for patients with multivessel disease to confirm or exclude balanced ischaemia, and low-cost baby cyclotrons will improve the cost-effective use of PET.

### Key points for PET

- PET is the clinical reference standard for the quantification of myocardial perfusion.
- Challenges of PET use include limited visual assessment of quantitative ^15^O-water PET and the lower spatial resolution of PET compared with CT or MRI perfusion imaging^29,30^.
- Small or ^13^N-ammonia cyclotrons, as well as the availability of ^18^F-labelled tracers, could improve the cost-effectiveness of cardiac PET imaging, as well as logistics and dissemination.
- Quantitative measures of myocardial ischaemia are provided with the addition of the measurement of viability and functional parameters during a single scan.
- Hybrid imaging combining PET perfusion imaging with coronary CT angiography data might provide comprehensive assessment, especially in patients with multivessel disease.

## MRI

### Role in assessment of ischaemic heart disease

Since its inception^85^, myocardial perfusion MRI has evolved into a clinical modality with excellent sensitivity and specificity for detecting myocardial ischaemia^86^. Most clinical myocardial perfusion MRI approaches are based on dynamic contrast-enhanced imaging using exogenous gadolinium-based contrast agents. The absence of ionizing radiation and the increasing availability of MRI expertise and equipment promote its routine use to guide clinical interventions. All components of a comprehensive MRI examination (that is, ventricular function and late gadolinium enhancement for viability and perfusion) add synergistic information to prognosis^87,88^. Long-term follow-up data have demonstrated a strong and independent predictive value of myocardial perfusion MRI for future major adverse cardiac events and death^89,90^. The MR INFORM study^91^ demonstrated that, among patients with stable angina and risk factors for coronary artery disease, the use of myocardial perfusion MRI was associated with a lower incidence of coronary revascularization than the use of FFR and was non-inferior to FFR with respect to major adverse cardiac events.

### Quantitative assessment of pathophysiology

**Fig. 5:**
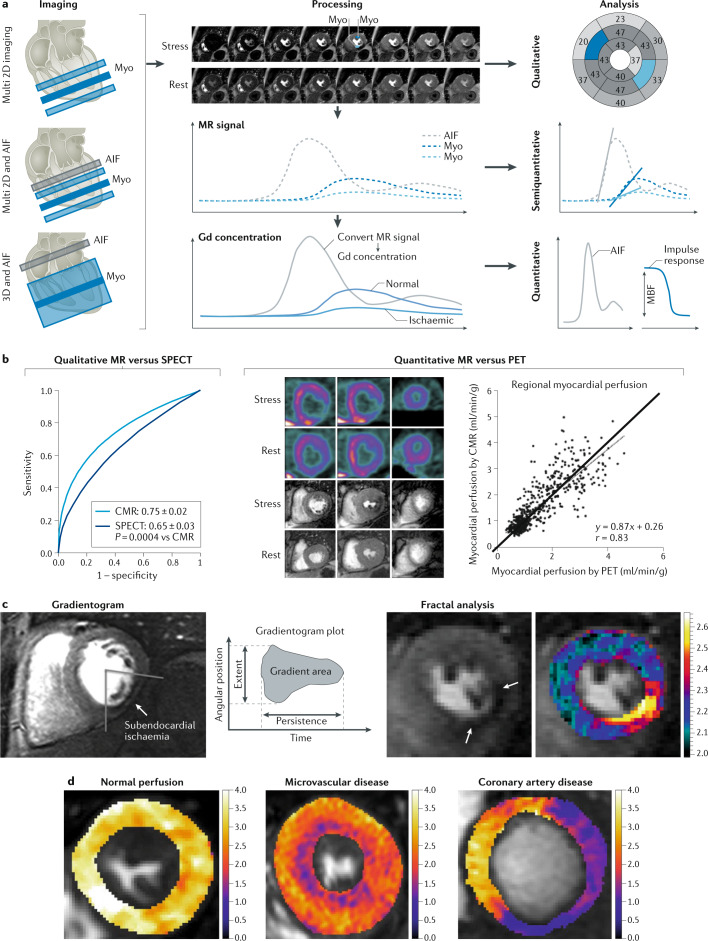
MRI for myocardial perfusion imaging. **a** | Imaging, processing and data analysis approaches in myocardial perfusion MRI. Standard multislice 2D MRI perfusion imaging yields image time series of dynamic myocardial signal change (Myo) during contrast agent bolus passage, allowing qualitative assessment of perfusion on the basis of eye-balling of hypoenhancing tissue (upper row). For semiquantitative analysis, the slopes of the dynamic magnetic resonance (MR) signal change during contrast passage are compared with the reduced slopes indicating ischaemic tissue (middle row). Using integrated arterial input function (AIF) measurements, which capture signal changes in the aortic outflow tract, and after conversion of dynamic MR signal changes to gadolinium (Gd) contrast agent concentrations, quantitative perfusion assessment can be performed, providing myocardial blood flow (MBF) values in units of millilitres per gram per minute. In conjunction with 3D MRI perfusion imaging, dynamic whole-heart coverage without slice gaps is possible (lower row). **b** | Comparison of qualitative cardiovascular MR perfusion analysis versus SPECT, demonstrating improved diagnostic performance of cardiovascular MR (CMR) versus SPECT in 425 patients in the MR-IMPACT II trial^108^ (left panel). Comparison of quantitative multislice 2D MR perfusion analysis versus PET in 41 patients (middle panel) and in 21 patients (right panel) showing a significant correlation between the modalities and providing evidence of the clinical utility of quantitative MR perfusion imaging^111,112^. **c** | Gradientogram analysis allows the measurement of radial extent, temporal persistence, area, peak and average intensity as well as strength of the transmural perfusion gradient (left panels) for detection of obstructive coronary artery disease^103^. Fractal analysis enables pathophysiological differentiation of epicardial disease and microvascular disease (arrows; right panels). **d** | Examples of fully quantitative high-resolution MRI perfusion maps. From left to right: example of an individual with normal stress perfusion, with homogeneous perfusion values ranging from 3 to 4 ml/g/min; example of a patient with angina, smooth epicardial coronary arteries and diffusely reduced stress perfusion values and more severely impaired subendocardial perfusion; and example of a patient with two-vessel coronary artery disease, with severe transmural impaired stress perfusion in the right and left circumflex coronary artery perfusion territories. Part **b** left panel adapted from ref.^108^, CC BY 2.0 (https://creativecommons.org/licenses/by/2.0/). Part **b** middle panel adapted with permission from ref.^111^, Elsevier. Part **b** right panel adapted from ref.^112^, CC BY 4.0 (https://creativecommons.org/licenses/by/4.0/). Part **c** left panels adapted with permission from ref.^103^, Wiley-VCH. Part **d** courtesy of C. Scannell, King’s College London, UK.

Detailed technical characteristics of MRI are listed in Table 1. Although mostly visual or semiquantitative assessment is used to identify ischaemia in the clinic^92^, quantitative approaches^93^ have gained momentum with technical advances in MRI data acquisition, reconstruction and processing^45,94^. Quantitative perfusion analysis provides incremental prognostic value over semiquantitative and qualitative data analysis, with an area under the receiver operating characteristic curve (AUC) of 0.85 versus 0.75 (ref.^88^). Figure 5a summarizes the technical approaches used for qualitative, semiquantitative and quantitative MRI perfusion. Although imaging is typically performed by acquiring three 2D sequences along the short axis in a dynamic fashion, the feasibility of dynamic 3D, whole-heart imaging has been shown^32^. The conversion of signal intensity to contrast concentration is an important step for quantitative assessment (Fig. 5a). This process involves nonlinear mapping and dual sequence or dual bolus approaches to derive AIF^95–97^. In practice, a model-free Fermi function model has been widely used, and provides high accuracy and precision^98^ (Fig. 5a). Compared with Fermi models, distributed parameter models offer increased sensitivity (78% versus 96%) and specificity (88% versus 92%) by providing additional insights into changes in the permeability surface area and interstitial volumes evaluated^99^. Automated voxel-wise quantification offers faster assessment than the manual quantification of perfusion but has similar accuracy^100^. However, absolute measures remain variable because they are tightly connected to the MRI sequence, and a lack of standardization exists between systems^41^. An automated method for AIF detection on the basis of pixel thresholding reduces analysis time and produces similar MBF values to those derived from manual AIFs^101^.

### Indications and clinical applications

MRI is less commonly used than SPECT but avoids ionizing radiation and allows the evaluation of patients with complex disease, who benefit from quantification of function, fibrosis and perfusion. Clinical indications for MRI are listed in detail in Fig. 2. Myocardial perfusion MRI has advanced substantially, with improved temporal resolution and the capacity to offer online automatic quantification in millilitres per gram per minute. The most important clinical application of absolute myocardial perfusion quantification is in patients with complex disease to address questions about multivessel disease, microvascular dysfunction^102^ or a combination of both^103^. Quantitative perfusion is also feasible in patients with heart failure and remodelled and thinned left ventricular walls, on the basis of the elevated spatial resolution allowed by cardiac MRI^104^. Methods for combined assessment of quantitative perfusion and left ventricular scar have been described^105^. T1 mapping can be used to differentiate between obstructive disease and microvascular dysfunction with higher accuracy than perfusion MRI and has produced similar mapping results in infarcted and ischaemic tissue^106^. In the large, single-centre CE-MARC study^107^, the AUC for perfusion MRI (0.89) was higher than that for SPECT (0.74) on the basis of visual assessment. This finding is in agreement with the secondary end points of the multicentre MR-IMPACT II study^108^ (Fig. 5b). However, quantitative MRI assessment in this study did not improve accuracy^109^, and the randomized, multicentre CE-MARC 2 study^110^ showed equal reductions in unnecessary invasive coronary angiography procedures using a diagnostic strategy on the basis of either MRI or SPECT. Compared with PET, quantitative perfusion MRI had a similar AUC of 0.83 for the detection of clinically significant coronary artery disease, but MRI perfusion values correlated only weakly with quantitative PET perfusion values, suggesting that further refinements are necessary^111^ (Fig. 5b). This refinement could be possible with perfusion mapping MRI, which has been shown to result in higher correlation with ^13^N-ammonia PET^112^ (Fig. 5b). However, most of these MRI studies used a limited spatial coverage of the left ventricle with only three cardiac short-axis slices, possibly resulting in missed diseases and limited spatial resolution with a slice thickness in the order of several PET voxel sizes. Another major challenge for the clinical application of MRI is its use in patients with cardiac devices, which nevertheless can be done safely when the pacing is changed to asynchronous mode for pacing-dependent patients or to demand mode for other patients, and tachyarrhythmia functions are disabled^113^. This approach might even allow cardiac MRI in patients with pacemakers or defibrillators, with interpretability not being limited by artefacts^31^.

### Future developments

Free-breathing perfusion MRI with subsequent motion correction will make the procedure easier for patients^114^. Improved detection of myocardial ischaemia is possible using the gradientogram analysis, which has been validated in comparison with FFR^115^ (Fig. 5c). Although most quantitative imaging has focused on myocardial ischaemia owing to coronary obstructions, microvascular dysfunction including impairment of vessel compliance will be the focus of comprehensive cardiac MRI protocols. Given that microvascular dysfunction occurs diffusively and has a patchy distribution^102^, it can be differentiated from ischaemia caused by coronary obstructions using fractal analysis^116^ (Fig. 5c).

High-resolution, pixel-wise automated quantification enables MBF to be measured on a pixel-by-pixel basis and microvascular disease to be distinguished from obstructive disease (Fig. 5d). The ability to visualize subendocardial ischaemia, limiting partial volume effects and spatial averaging, is one of the main technical advantages of perfusion MRI, contributing to the elevated sensitivity of this method for the detection of ischaemia. Machine learning applied to myocardial perfusion MRI might improve quantification and, together with under-sampling, might reduce acquisition and reconstruction times^27,117^.

In summary, cardiac MRI is a comprehensive test to investigate myocardial perfusion, fibrosis and function. Several approaches to automating image acquisition, reconstruction and assessment will facilitate the clinical implementation of myocardial perfusion MRI. As MRI does not require ionizing radiation, this modality is especially suited for use in young patients. On the basis of its characteristics, MRI should be considered in patients with complex disease who would benefit from imaging of function and fibrosis in addition to perfusion.

### Key points for perfusion MRI

- MRI avoids ionizing radiation and allows detailed characterization of myocardial tissue.
- Challenges associated with MRI application include the increased prevalence of pacemakers^31^, the limited spatial coverage of the left ventricle^32^ and the need for a method for adequate determination of arterial input function.
- Dedicated image-acquisition protocols (dual sequence) or contrast agent injection schemes (dual bolus) are required for quantitative MRI perfusion analysis.
- Automated voxel-wise quantification and dynamic 3D, whole-heart MRI could improve the detailed and complete assessment of myocardial ischaemia.
- Evaluation of patients with complex disease is improved by the quantification of function, fibrosis and perfusion, and fractal analysis might allow obstructive and microvascular disease to be differentiated.

## Echocardiography

### Role in assessment of ischaemic heart disease

**Fig. 6:**
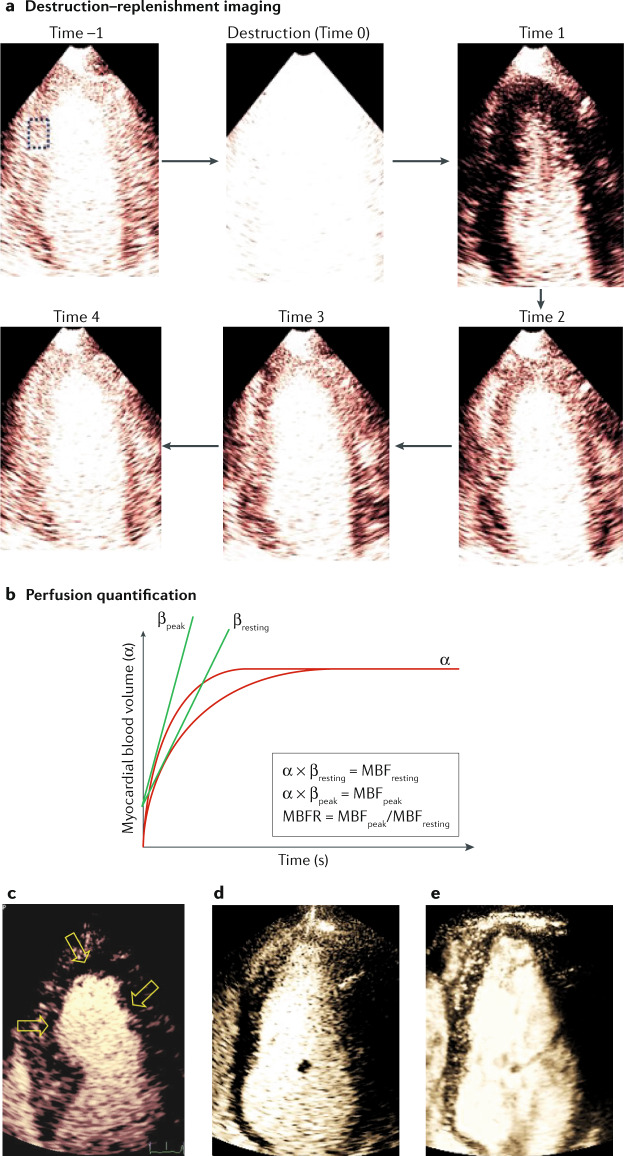
Contrast echocardiography for myocardial perfusion imaging. **a** | Principle of destruction–replenishment imaging with the destruction pulse causing a flash in the image intensity at time zero. **b** | Time–intensity analysis in the region of interest shown as a dashed square in part **a** to derive estimates of myocardial blood flow (MBF). **c** | Clinical image showing multiple perfusion defects in the mid-septum, apex and right wall (arrows). **d** | An example of suboptimal image quality, which is an important challenge of echocardiography. Other important challenges include time-consuming, manual region-of-interest analysis and the limited acoustic window. **e** | An example of high frame rate contrast echocardiography acquired in the same patient as in part **a**, demonstrating its potential to improve image quality. MBFR, myocardial blood flow reserve.

Echocardiography, like MRI, avoids ionizing radiation but can be performed at the bedside. Contrast echocardiography using microbubble tracers improves image quality and reproducibility in the assessment of global and regional left ventricular function^118,119^. The microbubbles consist of a gas core and typically a lipid or albumin shell. Ultrasound microbubbles remain entirely intravascular and can oscillate in resonance, producing the signals from individual microbubbles^120^. During infusion and steady state, signals emanating from microbubbles reflect the relative microvascular blood volume^121^. Microbubbles can be cleared from the myocardium by applying a series of high-power ultrasound pulses, and the myocardial replenishment is assessed by reverting to low-power imaging (Fig. 6a). This approach allows the myocardial blood velocity to be estimated as a semiquantitative measure of myocardial ischaemia^121^ both at rest and during stress (Fig. 6b). Echocardiography is the preferred technique in bedside situations, for instance in patients with suspected acute coronary syndrome, and can be useful in chronic myocardial ischaemia if good acoustic conditions are present. As collaterals supply blood to the myocardium, myocardial contrast echocardiography allows quantitative assessment of MBF subtended by an occluded artery. An association exists between collateral blood flow and myocardial viability after a recent myocardial infarction, as shown by quantitative myocardial contrast echocardiography^122^, which also accurately detects multivessel disease^123^. Using vasodilator stress, hyperaemic flow can be assessed in each of the 16 segments of the left ventricle, and CFR is calculated by dividing hyperaemic MBF by MBF during rest^124^. A good agreement has been shown between CFR measured by echocardiography and FFR, with the greatest discordance in symptomatic patients with normal FFR^125^. Echocardiography can also be used to assess viability in akinetic myocardial segments by assessing the microvasculature, which is present in viable but not necrotic tissue^126^.

### Quantitative assessment of pathophysiology

Detailed technical characteristics of echocardiography are listed in Table 1. Quantitative assessment of myocardium perfusion can be achieved through destruction–reperfusion imaging and analysis of the time–intensity curves from different regions of interest in the myocardium (Fig. 6). Quantitative myocardial perfusion at rest has been shown to predict viable myocardium after myocardial infarction and in chronic coronary artery disease^118^. MBF reserve assessed during hyperaemic echocardiography has been shown to be associated with the severity of coronary stenosis in patients with stable angina^118^. In a meta-analysis, MBF reserve had high accuracy for the prediction of flow-limiting coronary artery disease^118^ and could be used to determine the pathophysiological basis of microcirculatory angina^127^.

### Indications and clinical applications

Clinical indications for echocardiography are listed in detail in Fig. 2. Prospective studies including one randomized trial have investigated the use of contrast echocardiography for the detection of myocardial ischaemia (Fig. 6c) and its use for risk stratification of patients with known or suspected coronary artery disease, in both the acute and outpatient settings^128–132^. However, reliable quantification of myocardial perfusion remains elusive and sometimes even qualitative assessment can be challenging^133,134^. This limitation is first and foremost owing to variability in image quality (Fig. 6d). The common presence of noise and artefacts is an important factor that, in turn, affects the reproducibility of contrast echocardiography. This problem is particularly relevant for stress echocardiography, in which both high image quality and frame rates are required. In addition, limitations of the acoustic window can further diminish the signal-to-noise ratio. Echocardiography is ubiquitously available, is a bedside technique without ionizing radiation and is a preferred imaging technique among experienced operators in patients with acute chest pain.

### Future developments

An important challenge for echocardiography is the lack of automated quantification techniques and software. Although various physical and human factors affecting quantification are known and can be accounted for^135^, automated myocardial segmentation remains challenging owing to speckle noise and 3D deformations. Artificial intelligence solutions using machine learning for automated myocardial segmentation on contrast echocardiography are being developed^136^. These techniques promise automated and fast segmentation and perfusion quantification. High frame rate ultrasonography with up to 6,000 frames per second can be achieved through parallel data acquisition and digital beam forming. This approach offers image processing opportunities to increase the signal-to-noise ratio (Fig. 6c–e). High frame rate echocardiography can reveal the coronary microvasculature even without contrast but only in fairly shallow regions^137^. The feasibility of high frame rate contrast echocardiography for myocardial perfusion imaging in humans has been reported^33^. With this quantification, existing challenges might be addressed but its clinical utility needs further evaluation. In addition to high frame rate echocardiography, new 3D imaging^138^ might help to address the clinical application challenges discussed above.

In summary, echocardiography is a readily available bedside imaging test for which contrast-enhanced ultrasound applications using microbubbles have enabled semiquantitative measures of perfusion. High frame rate ultrasonography might overcome the major image quality issues that are often present during echocardiography, such as low signal-to-noise ratio and artefacts. Automated segmentation and 3D imaging will facilitate echocardiography. On the basis of its characteristics, echocardiography should be considered when bedside imaging is required.

### Key points for echocardiography

- Echocardiography is most commonly applied for cardiac function imaging but is increasingly also being used for the assessment of myocardial perfusion and can be performed at the bedside.
- The challenges of echocardiography include the common presence of noise and artefacts, lack of reproducibility, variable image quality and time-consuming manual analysis^33^.
- High frame rate echocardiography allows the estimation of myocardial blood velocity as a semiquantitative measure of myocardial ischaemia with improved image quality.
- Artificial intelligence solutions using machine learning for automated myocardial segmentation promise automated and fast segmentation and perfusion quantification.
- Evaluation of patients with suspected acute coronary syndrome at the bedside is the primary strength of echocardiography when performed by experienced operators.

## Cardiac CT

### Role in assessment of ischaemic heart disease

Cardiac CT is widely available with widespread clinical application for noninvasive coronary angiography and has a class I (level of evidence B) recommendation in the 2019 European guidelines, together with functional imaging, for use in individuals with a low-to-intermediate clinical likelihood of coronary artery disease^4^. However, stenosis grading by CT is limited in its capacity to predict ischaemia^139^, and CT perfusion imaging is not frequently used. For appropriate spatial coverage, >64 CT detector rows are recommended, while 256-row and 320-row CT provides whole-heart coverage in one heartbeat. For myocardial CT perfusion imaging, static imaging either with or without a vasodilator stressor (such as adenosine) is used, whereas dynamic (4D) imaging allows the quantification of MBF but requires multiple scans over time. Dynamic CT perfusion imaging might provide the greatest clinical value but challenges with this modality include increased radiation dose, motion artefacts and image quality limitations^140^. A meta-analysis showed higher sensitivity (0.85 versus 0.72) but lower specificity (0.81 versus 0.90) for dynamic compared with static CT perfusion imaging^141^. The greatest role of CT in relation to myocardial ischaemia might be in stenosis and plaque quantification.

### Quantitative assessment of pathophysiology

**Fig. 7:**
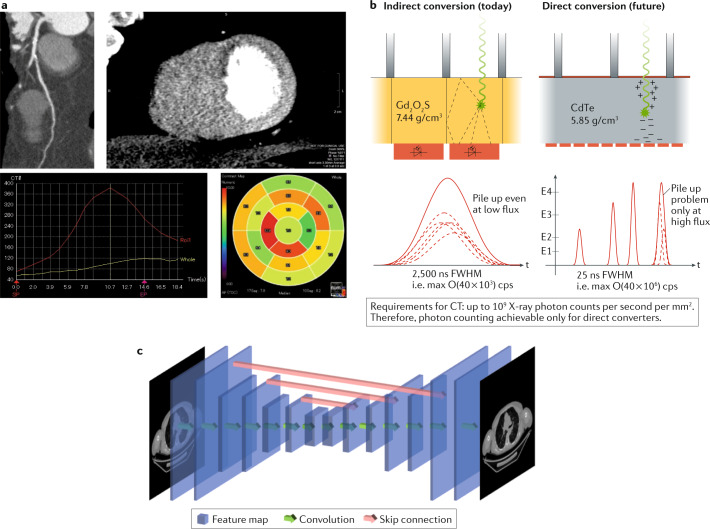
CT for myocardial perfusion imaging. **a** | CT angiography showing a coronary stenosis in the left anterior descending coronary artery (top left) and the corresponding anterior myocardial perfusion defect during adenosine stress (top right)^49^. Time attenuation curve (bottom left) and left ventricular polar map of absolute myocardial blood flow (bottom right). **b** | Photon-counting energy-selective X-ray detectors show promise in improving quantification of tissue densities in CT and increased contrast-to-noise ratio (compared with standard CT perfusion). **c** | Machine learning using convolutional deep neural networks can generate high-quality images from low-dose CT acquisition and reduce image artefacts. FWHM, full-width at half-maximum.

Detailed technical characteristics of CT are listed in Table 1. Advantages of CT include its high spatial resolution and the linear relationship between image attenuation, measured in Hounsfield units, and myocardial contrast agent concentration. Therefore, myocardial perfusion can, in principle, be measured quantitatively (absolute MBF)^142^ (Fig. 7a) and/or semiquantitatively (static imaging)^143,144^.

Furthermore, microvascular disease in addition to collateral circulation specifically in patients with occluded vessels can also be evaluated^145,146^. Although absolute quantification of ischaemia is possible, the added value over semiquantitative assessment is uncertain^147^. Current CT scanners are challenged by beam-hardening artefacts, limited temporal resolution resulting in cardiac motion artefacts and low contrast-to-noise ratios. Beam-hardening artefacts can be reduced using dedicated correction methods^34–36^. Temporal resolution can be improved by a higher rotation speed, dual-source CT or post-acquisition algorithms that estimate motion in order to compensate for it during image reconstruction^37^. Image acquisition during systole has the potential to improve CT perfusion image analysis, whereas β-blockers, which are often required for coronary CT angiography, might diminish the subsequent intended effects of vasodilator stressors^148^.

### Indications and clinical applications

Cardiac CT has undergone exponential growth in the past decade, and clinical indications are listed in detail in Fig. 2. Coronary CT angiography is included in clinical guidelines as a diagnostic tool in stable patients with chest pain and an intermediate pretest probability of obstructive coronary artery disease^1,3,4^. However, in patients with heavily calcified or stented coronary lesions, the addition of CT perfusion to CT angiography improves diagnostic accuracy from 71% to 87%^149^. Using combined CT angiography and CT perfusion might therefore reduce the number of unnecessary invasive coronary angiography procedures performed^150,151^. To identify patients in whom coronary revascularization might relieve myocardial ischaemia, myocardial CT perfusion imaging^147^ and computational fluid dynamics modelling to estimate FFR by CT have been investigated^152,153^. The international CORE320 study^154^ was the first to demonstrate that the combination of static CT myocardial perfusion imaging and coronary CT angiography can correctly identify coronary stenosis associated with myocardial ischaemia by SPECT, and this finding has been corroborated in a meta-analysis^155^. Initial experience suggests that the combination of CT myocardial perfusion imaging and FFR estimated by CT can provide an even higher diagnostic accuracy, with an improvement in the AUC from 0.78 to 0.85 (ref.^156^). Importantly, computational fluid dynamics modelling to estimate FFR relies on mathematical assumptions and equal responses by the microcirculation to vasodilators. Moreover, and in contrast to invasive FFR, noninvasively estimated FFR does not account for collateral flow in the same manner^157^. To what extent the implementation of CT–FFR will translate into improved patient outcomes still needs to be elucidated. Of note, a meta-analysis showed that CT perfusion has a higher specificity than CT–FFR (86% versus 78%)^141^. The randomized CATCH-2 trial^158^ demonstrated that the addition of CT myocardial perfusion imaging to coronary CT angiography alone safely reduces the need for invasive examination and treatment (Fig. 7a). The CRESCENT II trial^159^ demonstrated the benefit of a tiered CT protocol with limited use of CT perfusion only if CT angiography shows a ≥50% stenosis, which led to more appropriate revascularization indications than functional testing (88% versus 50%). In a two-centre study, semiquantitative static CT myocardial perfusion imaging achieved a similar performance to perfusion MRI compared with the reference standard of invasive angiography and SPECT^160^. This finding was supported in a meta-analysis, which showed similar negative likelihood ratios for perfusion imaging using PET, MRI or CT of 0.14, 0.14 and 0.12, respectively^73^. Importantly, in the 2-year follow-up of the CORE320 study^161^, the combination of CT angiography and semiquantitative CT perfusion showed similar accuracy in predicting cardiovascular events to invasive coronary angiography and SPECT combined. In another single-centre study, MBF derived from dynamic CT perfusion had independent predictive value, with a hazard ratio of 5.7 when adjusted for obstructive coronary artery disease on CT angiography^162^.

### Future developments

Two-compartment analysis might reduce underestimation of MBF by CT^163,164^, and the semiquantitative transmural perfusion ratio might improve diagnosis of myocardial ischaemia compared with using the MBF^165^. Low kilovolt scanning (70–80 kV) enables a substantial reduction in radiation dose, while increasing contrast-to-noise ratio^166^. New photon-counting energy-selective X-ray detectors are being developed for improved contrast and reduced radiation dose^167^ (Fig. 7b). Algorithmic solutions will further improve contrast and reduce noise^168^. Four-dimensional, non-rigid motion correction will allow visual interpretation and pave the way for quantitative assessment of the 2–3 billion voxels acquired during dynamic CT perfusion imaging^169^. Convolutional neural networks will reduce artefacts (Fig. 7c) by predicting high-quality images from low-dose CT^170,171^. Sparse-view CT is a futuristic way of reducing radiation dose in which the dose-emitting source is masked at different angular positions during gantry rotation. The resulting structured CT image artefacts might be corrected using deep neural networks^26^, and accurate scatter correction might become possible using deep learning, which is much faster than using Monte Carlo simulations^172^.

In summary, CT perfusion is rarely used but theoretically well suited for quantification of myocardial perfusion in addition to its capacity noninvasively to exclude obstructive coronary stenosis and to characterize coronary atherosclerosis^173^. Low kilovolt scanning, new CT detector technology and algorithmic solutions are promising approaches to reducing CT radiation dose and image artefacts. On the basis of these characteristics, the greatest value of CT in relation to myocardial ischaemia is for the combined assessment of stenosis and atherosclerosis.

### Key points for CT

- CT perfusion imaging is not frequently used, whereas CT angiography offers highly accurate coronary angiography.
- Challenges of cardiac CT include limited temporal resolution, beam and scatter artefacts, radiation dose and low contrast-to-noise ratios^34–37^.
- CT systems with increased rotation speed, photon-counting energy-selective X-ray detectors and advanced machine learning technology, as well as two-compartment analysis could overcome the technological challenges.
- The combined assessment of stenosis and characterization of atherosclerosis in relation to myocardial ischaemia promises the greatest clinical value but requires testing in trials.
- Evaluation of patients with insufficient angiographic image quality or borderline stenosis on coronary CT angiography might be improved with the use of CT for myocardial ischaemia.

## Invasive coronary flow and pressure

### Role in assessment of ischaemic heart disease

Invasive coronary physiology techniques have an increasing role in the clinical assessment of stenosis-induced myocardial ischaemia. Their availability during catheterization allows informed decision-making about coronary revascularization directly in the catheterization laboratory^174^. Invasive coronary flow and pressure measurement is recommended in clinical guidelines to guide decision-making in the absence of noninvasive stress testing results^1^. To enable better decision-making in these situations, the advantages and limitations of coronary physiology techniques in clinical practice must be recognized.

### Quantitative assessment of pathophysiology

**Fig. 8:**
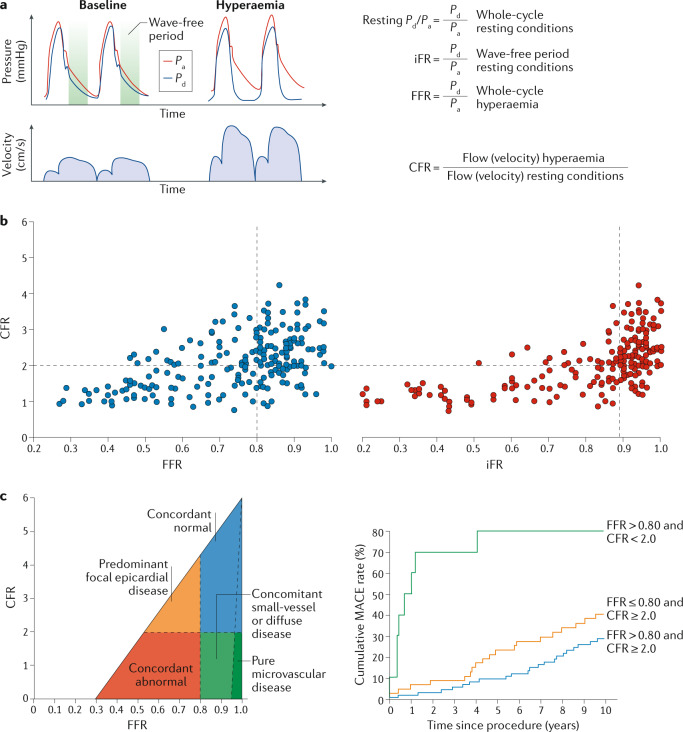
Invasive coronary flow and pressure measurement. **a** | Representation of the ratio of resting distal coronary pressure (*P*_d_) to aortic pressure (*P*_a_), instantaneous wave-free ratio (iFR), fractional flow reserve (FFR) and coronary flow reserve (CFR) calculation from invasively assessed coronary pressure or flow measurement. **b** | Agreement between coronary pressure (FFR or iFR) and CFR measurement. Discordance between FFR and CFR occurs in 30–40% of individuals, whereas better agreement can be observed between iFR and CFR. **c** | Interpretation of combined FFR and CFR measurement and its effect on clinical outcome^193^. Four main quadrants can be identified by the clinical cut-off values for FFR and CFR, indicated by the dashed lines. Patients in the upper right area (blue) have normal FFR and CFR; patients in the lower left area (red) have abnormal FFR and CFR; patients in the upper left area (orange) have abnormal FFR and normal CFR; and patients in the lower right area (green) have normal FFR and abnormal CFR, which indicates predominant microvascular involvement or diffuse coronary artery disease. Patients in the small dark green region in the lower right have an FFR close to 1 and an abnormal CFR, indicating sole involvement of the coronary microvasculature. The prognostic value of FFR and CFR in terms of major adverse cardiovascular events (MACE) is shown on the right. Part **c** adapted with permission from ref.^193^, van de Hoef, T. P. et al. Physiological basis and long-term clinical outcome of discordance between fractional flow reserve and coronary flow velocity reserve in coronary stenoses of intermediate severity. *Circ. Cardiovasc. Interv*. **7**(3), 301–311 (https://www.ahajournals.org/journal/circinterventions).

Detailed technical characteristics of invasive coronary flow and pressure measurement are listed in Table 1. The most widely adopted invasive approach to pathophysiology is (myocardial) FFR, which is calculated as the ratio of mean distal coronary pressure to mean aortic pressure during pharmacologically induced coronary vasodilatation (Fig. 8a). The aim of FFR is to quantify the impairment of maximal coronary flow induced by a stenosis from invasive coronary pressure measurement^175^. FFR-guided coronary interventions substantially reduce the need for revascularization procedures compared with decision-making by visual assessment alone and leads to similar long-term clinical outcomes^176^. However, two important issues remain. First, the adoption of FFR guidance remains limited to <6% of patients in most countries globally, with only a few exceptions^38^. This situation is in stark contrast to clinical guidelines, which advocate the widespread use of invasive coronary measurements. Furthermore, noninvasive ischaemia imaging tests are used in only 64% of all patients before coronary angiography^150,151^. Second, the prognostic value of FFR in patients with stenoses and an abnormal FFR is limited, with 27% of patients who are managed by medical therapy alone having a cardiovascular event within 5 years^39^. This finding calls into question the clinical practice guidelines advocating revascularization in all vessels with an abnormal FFR^1^. The limited adoption of FFR is related to practical and economic issues as the procedure is both time-consuming and costly. Some of these problems can be addressed by the use of simpler measurements, such as the instantaneous wave-free ratio (iFR) or resting distal coronary-to-aortic pressure ratio (*P*_d_/*P*_a_). iFR is defined as the ratio of distal coronary pressure to aortic pressure at a distinct time in cardiac diastole, termed the wave-free period (Fig. 8a), in resting, non-vasodilated conditions^177^. The diagnostic efficiency of FFR and iFR for haemodynamically significant coronary stenoses is similar to that of SPECT or PET^38^. Moreover, an iFR-guided revascularization strategy has been documented to provide equivalent clinical outcomes to those of an FFR-guided strategy in two large randomized clinical trials, where fewer coronary revascularization procedures were indicated in the iFR-guided treatment groups^178,179^.

Importantly, pressure-based measurements provide an estimate of stenosis-induced flow impairment, but they are different from coronary flow or CFR, which determine ischaemia. CFR is defined as the ratio of coronary flow during pharmacological vasodilatation to flow at rest (Fig. 8a), representing the reserve capacity of the coronary circulation to increase flow in response to increased demand. A lower CFR is linked to symptoms of ischaemia and is predictive of myocardial infarction and death. A challenge is that coronary pressure-derived estimates of flow impairment do not correlate with measured CFR in 30–40% of patients^132,133^ (Fig. 8b). This situation arises because physiology dictates that an increase in coronary flow leads to lower (abnormal) FFR but higher (normal) CFR and vice versa^180^. This observation suggests that combined FFR and CFR is better for identifying the individual pathophysiology and predicting clinical outcomes^132^ (Fig. 8c).

FFR and iFR are considered to estimate the effect of the stenosis on distal MBF, thereby taking into account the effect of collateral flow to the myocardium of interest. For FFR, coronary FFR can be calculated, which is the FFR in the coronary artery when excluding the effect of collateral flow. However, myocardial FFR has been studied in clinical outcome trials and is used in daily clinical practice. Nonetheless, coronary collateral flow can be assessed directly using invasive coronary pressure and flow measurements in several ways. Coronary wedge pressure, which is the distal coronary pressure obtained during occlusion of the epicardial coronary artery to prevent antegrade coronary flow, is a simple marker of collateral function. Furthermore, collateral blood flow can be quantified in more detail using either flow velocity or coronary pressure measurements. The Doppler velocity-derived collateral flow index (CFI) is calculated as the intracoronary flow velocity integral (CFVI) during balloon occlusion as a fraction of CFVI during coronary patency. The coronary pressure-derived CFI (CFIp; also called collateral FFR) is calculated as the coronary wedge pressure minus venous back pressure as a fraction of the aortic pressure minus venous back pressure. When both Doppler velocity and coronary pressure measurements are performed, the total collateral resistance can also be calculated, as well as the individual resistances in the components of the collateral pathway. CFI and CFIp are the most applicable techniques, and values of >0.30 are generally considered to indicate adequate collateral artery function, substantiated by the absence of angina or electrocardiogram changes during balloon occlusion^181^.

### Indications and clinical applications

Clinical indications for invasive approaches are listed in detail in Fig. 2. In patients with a high likelihood of invasive treatment being required, invasive coronary flow and pressure measurement is well suited to guide treatment decision-making, with FFR having achieved greatest acceptance but limited clinical uptake^38^. This situation might be improved using less complex invasive measurements, such as iFR, which compared with an FFR-guided strategy leads to fewer revascularizations while providing equivalent 1-year clinical outcomes^178,179^. Resting *P*_d_/*P*_a_ has so far been investigated only in diagnostic studies and has shown similar accuracy to iFR^182^. Considering the limitations of coronary pressure-based techniques, the decision to revascularize should optimally be on the basis of the combination of imaging ischaemia tests and coronary pressure measurement, the results of which should be interpreted in relation to the symptoms of the individual patient to determine the risk–benefit ratio of a coronary intervention.

### Future developments

The suboptimal discriminative value of FFR can be addressed by the (additional) direct measurement of coronary flow, instead of its estimation from coronary pressure measurement^180^. This approach is currently being evaluated in a multicentre setting in the DEFINE-FLOW study^183^. Meanwhile, it is important to realize that the current technology for direct flow measurement is fairly outdated and technically demanding, and substantial technical revisions are expected shortly. However, current ischaemia-guided or coronary pressure-guided coronary interventions are already superior to angiography-based decision-making alone^176,178,179^. Artificial intelligence, mainly in the form of machine learning and intelligent algorithms, is increasingly being recognized as a tool to aid invasive diagnostics^184^ and to improve the safety and accuracy of invasive physiology techniques. Examples are the automated interpretation of aortic pressure traces and iFR pullback traces to identify inaccurate pressure readings that might influence FFR and/or iFR values or their interpretation and affect decision-making.

In summary, FFR measurements can substantially reduce the need for revascularization compared with visual assessment alone, without impairing long-term clinical outcomes. However, the limited uptake of invasive measurement of FFR in clinical practice is related to the complexity of the procedure, which can be overcome using measurement of iFR and *P*_d_/*P*_a_. The combined measurement of pressure and flow should have the highest discriminative capacity and prognostic value but requires further testing. On the basis of their characteristics, invasive measurements have the greatest value in patients with a high likelihood of requiring invasive treatment.

### Key points for invasive coronary flow and pressure measurement

- Invasive catheter-based methods can be used to measure coronary flow and pressure.
- Challenges of invasive coronary flow and pressure measurements include the diagnostic and prognostic characteristics of FFR, the complexity of the procedure and its limited uptake in clinical practice (used in <6% of patients^38,39^).
- iFR and resting *P*_d_/*P*_a_ are simpler than FFR but have similar prognostic and diagnostic value and might improve the clinical uptake of coronary pressure measurements.
- The combined assessment of CFR and FFR promises the greatest discriminative value for individual pathophysiology but requires testing in trials.
- In patients with a high likelihood of requiring invasive treatment, invasive coronary flow and pressure measurement is well suited to guide treatment decision-making.
- Invasive coronary pressure and flow measurements allow coronary collateral function to be quantified.
- Artificial intelligence might further improve the accuracy and interpretation of coronary pressure readings and could improve coronary flow assessment.

## Conclusions

Current cardiac imaging modalities offer widespread utility to assess myocardial ischaemia quantitatively, without the risks of invasive investigation. Among the myocardial perfusion imaging tests, PET is the reference standard for quantification, and SPECT is most commonly used. Although MRI is less commonly used than SPECT, MRI avoids ionizing radiation, as does echocardiography. Echocardiography has similar advantages to those of MRI and can be performed at the bedside. CT is not frequently used for perfusion imaging but offers coronary angiography including assessment of plaque burden and can provide a geometric framework for model-based estimation of CT–FFR. Intracoronary measurements of pressure and flow velocity provide comprehensive haemodynamic information to guide invasive treatment. All six ischaemia imaging techniques have distinct advantages and disadvantages (Table 1).

The diagnostic potential of these imaging techniques is substantially improved by technical advances that can yield quantitative measures of myocardial perfusion. Dedicated cardiac SPECT cameras have emerged for improved sensitivity and resolution at lower doses of radiation, and dynamic imaging enables compartmental modelling and thereby provides absolute measures of myocardial ischaemia. Small cyclotrons and the availability of ^18^F-labelled PET tracers could improve logistics, and hybrid PET–CT might provide comprehensive assessment, especially in patients with multivessel disease. Automated voxel-wise perfusion quantification of whole-heart MRI could improve the assessment of myocardial ischaemia to facilitate the evaluation of patients with complex disease and the differentiation between obstructive and microvascular disease. High frame rate echocardiography allows the estimation of myocardial blood velocity with improved image quality, and machine learning can be used for automated and fast myocardial segmentation. CT with increased rotation speed, photon-counting detectors and machine learning could overcome temporal resolution challenges, beam and scatter artefacts, and low contrast-to-noise ratios of CT perfusion imaging for use in patients with borderline coronary stenosis on CT angiography. Invasive coronary measurements of iFR and resting *P*_d_/*P*_a_ is simpler than measurement of FFR but have similar prognostic and diagnostic value and might improve the so-far limited uptake of coronary pressure measurements. The combined assessment of CFR and FFR provides the greatest promise in discriminating individual pathophysiologies.

No single quantitative perfusion imaging technique is best for all types of patient, for all disease stages or at all clinical centres, as usage also depends on the availability of local expertise. Using a Delphi consensus process, we have identified the most appropriate clinical scenario for each diagnostic test for myocardial ischaemia (Fig. 2). SPECT with the use of new detectors allows quantification of MBF and is also suited to quantification in patients with a high BMI. PET should be the primary test in patients with multivessel disease to confirm or exclude balanced ischaemia. MRI allows the evaluation of patients with complex disease who would benefit from imaging of cardiac function and myocardial fibrosis in addition to perfusion. Echocardiography is the preferred technique for assessing acute coronary syndrome in bedside situations. CT has greatest value for combined quantification of stenosis and characterization of atherosclerosis in relation to myocardial ischaemia. In patients with a high likelihood of requiring invasive treatment, invasive coronary flow and pressure measurement is well suited to guide treatment decision-making.

All imaging modalities share the requirement to take coronary pathophysiology into account in the interpretation of noninvasive perfusion imaging and invasive coronary pressure and flow data. Quantification of myocardial perfusion by imaging can provide accurate measures of the burden of ischaemia to determine individualized treatment strategies. This assessment is very important because the ORBITA study^134^ showed that a much more comprehensive, pre-catheterization, quantitative, multimodality imaging strategy is required in patients with suspected ischaemia. This approach helps to identify the most appropriate treatment strategy with or without coronary revascularization and might translate into lower morbidity and mortality. Therefore, in the time of personalized medicine, perfusion imaging provides robust, quantitative assessment of myocardial ischaemia that can be used to guide individual risk stratification and direct treatment decision-making. Future studies will incorporate the emerging computational support of machine learning to benefit complex decision-making about the need for coronary revascularization.

## Supplementary information

Supplementary Video 1 | **Computational fluid dynamics simulation of coronary blood flow.** The video shows a Gd-DOTA bolus as it is transported by pulsatile blood flow along the epicardial arteries^50^. The video was generated on the basis of computational fluid dynamics simulations using the 3D geometry of a porcine arterial tree. For better visualization of inhomogeneous transport effects, a much shorter bolus was used for this simulation than would be used in humans. Still images from the video are shown in FIG. 1d and were previously published in ref.^50^.

Supplementary Video 2 | **Magnified view of a portion of Supplementary Video 1.** The video shows the complex dynamics of a Gd-DOTA bolus as it is transported through the epicardial vessels^50^. Note that this video is a 2D visualization of a 3D geometry. For better visualization of inhomogeneous transport effects, a much shorter bolus was used for this simulation than would be used in humans. Still images from the video were previously published in ref.^50^.
